# Diagnostic and mechanistic roles of miR-5584-5p in endometriosis via the *FZD2*-mediated Wnt/β-catenin EMT axis

**DOI:** 10.1186/s41065-026-00732-4

**Published:** 2026-08-21

**Authors:** Wandi Zhang, Min Zhang, Han Lu, Wanli Ji

**Affiliations:** 1 Department of Reproductive Medicine, The First Affiliated Hospital of Zhengzhou University, Zhengzhou, 450000 China; 2 Department of Obstetrics and Gynecology, Suzhou BenQ Medical Center, The Affiliated BenQ Hospital of Nanjing Medical University, Suzhou, 215004 China; 3 Department of Obstetrics and Gynecology, Guangdong Corps Hospital of People’s Armed Police, Guangzhou, 510507 China; 4 Department of Gynaecology and Obstetrics, Nanjing Luhe People’s Hospital, No. 28, Yan’an Road, Luhe District, Nanjing, 211599 China

**Keywords:** miR-5584-5p, Endometriosis, *FZD2*, Wnt/β-catenin signaling, Diagnostic biomarker

## Abstract

**Background:**

The early detection of endometriosis (EM), a significant cause of dysmenorrhea, is essential for effective clinical management. This study sought to determine the functional role of miR-5584-5p in EM and the mechanistic involvement of its target, *FZD2*, in disease progression.

**Methods:**

This case-control study enrolled 217 dysmenorrhea patients (107 EM cases and 110 controls). Ishikawa cells with modulated miR-5584-5p and *FZD2* expression were used to assess cell proliferation, migration, and invasion. The miR-5584-5p/*FZD2* interaction and Wnt/β-catenin transcriptional activity were validated by dual-luciferase and TOP/FOP flash reporter assays.

**Results:**

Serum miR-5584-5p was significantly downregulated in EM patients, demonstrating high diagnostic accuracy (AUC = 0.903) and acting as an independent protective factor. In vitro, miR-5584-5p overexpression suppressed Ishikawa cell proliferation, migration, and invasion, concurrently inhibiting Wnt/β-catenin transcriptional activity and the epithelial-mesenchymal transition (EMT) axis. *FZD2* was confirmed as a direct target of miR-5584-5p. Crucially, *FZD2* overexpression partially reversed the inhibitory effects of miR-5584-5p on malignant cellular phenotypes and restored Wnt/β-catenin signaling.

**Conclusion:**

Serum miR-5584-5p serves as a valuable potential diagnostic biomarker for EM. Functionally, it attenuates endometrial epithelial cell aggressiveness by targeting *FZD2* and suppressing the Wnt/β-catenin-EMT axis. Future studies utilizing primary cells and in vivo models are warranted to validate these preliminary mechanisms.

**Supplementary Information:**

The online version contains supplementary material available at https://doi.org/10.1186/s41065-026-00732-4.

## Background

Dysmenorrhea is the most common gynecological symptom among women of childbearing age, and Endometriosis (EM) is one of its significant causes [1]. Accurately identifying patients who have or will develop EM from the large dysmenorrhea population remains a clinical challenge. Furthermore, EM is a well-established risk factor for endometrial cancer [2]. Therefore, understanding the molecular events driving the progression from dysmenorrhea to EM is vital for early diagnosis and intervention.

MicroRNAs (miRNAs) are crucial post-transcriptional regulators, and their dysregulation is frequently implicated in EM pathogenesis [3]. A previous study identified significantly low expression of hsa-miR-5584-5p in both ectopic and eutopic endometrial tissues from EM patients [4], suggesting that this miRNA may be involved in EM pathogenesis. The core pathological processes of EM include abnormal proliferation, invasion, and migration of ectopic endometrial cells, sharing features with malignant cells [5, 6]. Within this context, epithelial-mesenchymal transition (EMT) enables cells to acquire invasive properties [7]. The Wnt/β-catenin signaling pathway, a known EMT promoter, is prominently associated with both EM and endometrial cancer [8, 9].

*FZD2*, a member of the Frizzled family, is a component of the Wnt/β-catenin pathway involved in receiving Wnt signals and initiating downstream cascades [10, 11]. Studies have shown that *FZD2* is upregulated in various cancers (such as esophageal cancer) and may contribute to EMT and tumor invasion [11]. However, the specific role of *FZD2* in the early stages of EM, particularly in the transition from dysmenorrhea to EM and its potential for malignant progression, remains unclear.

Given the downregulation of miR-5584-5p in EM tissues reported previously, we hypothesized that reduced miR-5584-5p expression might serve as a molecular marker to identify EM patients among women with dysmenorrhea. In this study, we aimed to evaluate the diagnostic potential of serum miR-5584-5p in a cohort of dysmenorrhea patients with and without EM, and to explore its functional role in endometrial epithelial cells using an in vitro model. We acknowledge that miRNA regulatory networks are complex, and while this study focuses on the miR-5584-5p/*FZD2* axis, other targets may also contribute to the observed effects.

## Methods

### Study subjects

This study enrolled 217 patients with dysmenorrhea between 2022 and 2024. Based on laparoscopic findings, they were categorized into an EM group (*n* = 107) and a non-EM group (*n* = 110). The study received approval from the Ethics Committee of Guangdong Corps Hospital of People’s Armed Police and adhered to the ethical principles of the Declaration of Helsinki. Written informed consent was obtained from all participants.

The inclusion criteria were: (1) reproductive age (18–45 years); (2) clear and cyclical dysmenorrhea symptoms; (3) EM group patients confirmed by laparoscopy and postoperative pathology; (4) non-EM group patients confirmed by clinical evaluation and imaging examinations (e.g., pelvic ultrasound) to exclude EM and other pelvic organic lesions; (5) complete clinical data.

Exclusion criteria included: (1) concomitant other gynecological diseases such as adenomyosis, pelvic inflammatory disease, or ovarian tumors; (2) use of hormonal medications (e.g., GnRH-a, oral contraceptives) within the past 3 months; (3) pregnancy or lactation; (4) severe cardiovascular, cerebrovascular, hepatic, renal, or other systemic diseases, or autoimmune diseases; (5) history of major pelvic or abdominal surgery (except cesarean section).

### Patient information and serum sample collection

During clinical consultation, this study systematically collected basic information and clinical data from all subjects. Following collection, morning fasting venous blood samples (5 mL) were allowed to clot at room temperature for 30 min and then centrifuged at 3000 rpm under 4 °C for 10 min. The separated serum was aliquoted into 500 µL cryotubes and stored in a ‑80 °C ultra-low temperature freezer. All analyses were performed using samples subjected to only one freeze-thaw cycle.

### Cell culture and transfection

The human endometrial adenocarcinoma cell line Ishikawa (ATCC, USA) was used as an in vitro model for endometrial epithelial cells, following the approach described by Tang et al. [12]. Cells were cultured in RPMI-1640 medium (Thermo Fisher Scientific, USA) supplemented with 10% fetal bovine serum (Excel Bio, China) and 1% penicillin-streptomycin (Beyotime, China) at 37 °C in a 5% CO2 humidified incubator. Transfections were performed using Lipofectamine 3000 (Invitrogen, USA). Cells were divided into five groups: negative control (NC), mimic NC, miR-5584-5p mimic, inhibitor NC, and miR-5584-5p inhibitor. For rescue experiments, OE-NC or OE-*FZD2* plasmids were co-transfected with the miR-5584-5p mimic. Transfection efficacy was confirmed by qRT-PCR at 48 h. All experiments were performed in triplicate (*n* = 3).

### qRT-PCR

Total RNA was extracted with TRIzol™ Reagent (Thermo Fisher Scientific, USA) from cultured cells or from 200 µL serum samples. For serum RNA extraction, samples were processed strictly on ice to minimize degradation. TRIzol™ Reagent was utilized as it rapidly denatures endogenous RNases during the lysis step. The detected *FZD2* mRNA in serum is likely protected within circulating exosomes or RNA-protein complexes, as previously described [13, 14]. To guarantee the technical reliability of detecting cell-free mRNA, strict quality control was enforced before reverse transcription: RNA integrity was confirmed by the presence of clear 28 S/18S bands without severe smearing on 1.5% agarose gel electrophoresis, and adequate purity was ensured via a microspectrophotometer (A260/A280 ratios between 1.8 and 2.0). cDNA was synthesized from qualified RNA using the PrimeScript™ RT Reagent Kit with gDNA Eraser (Takara, Japan) on a T100™ Thermal Cycler (Bio-Rad, USA). qPCR was conducted on a QuantStudio™ 5 System (Applied Biosystems, USA) with SYBR^®^ Premix Ex Taq™ II (Takara, Japan) under the following conditions: 95 °C for 30 s; 38 cycles of 95 °C for 5 s and 60 °C for 30 s. Melt curve analysis confirmed amplification specificity. Gene expression (miR-5584-5p, *FZD2*, *MYC*, *CCND1*, *AXIN2*, *CDH1*, *VIM*, *SNAI1*) was normalized to GAPDH (mRNA) or U6 (miR-5584-5p) and quantified via the 2^–ΔΔCT^ method. Each assay included three technical and three biological replicates (*n* = 3).

### CCK-8 assay

The proliferative capacity of Ishikawa cells was evaluated with a CCK-8 assay. Cells in the logarithmic growth phase were seeded into 96-well plates at a density of 3 × 10³ cells per well and subjected to transfection. Following transfection, cell viability was measured at 0, 24, 48, and 72 h by incubating with 10 µL CCK-8 for 2 h at 37 °C and reading the absorbance at 450 nm. Results were plotted as a growth curve relative to the 0-hour baseline. All functional assays were independently repeated three times (*n* = 3).

### Transwell assay

Cell migration and invasion were evaluated using Transwell chambers (8 μm pore size, Corning, USA). For the invasion assay, the upper chamber was precoated with 50 µL of Matrigel (BD Biosciences, USA), diluted at a 1:8 ratio using serum-free medium, and allowed to solidify at 37 °C for 4 h. After transfection, cells were collected and resuspended in serum-free medium (HyClone, USA). A total of 5 × 10⁴ cells suspended in 200 µL were seeded into the upper chamber, with the lower chamber filled with 500 µL of complete medium containing 10% FBS (Excel Bio, China) as a chemoattractant. After a 24-hour incubation at 37 °C, non-migrated cells were removed from the upper chamber with a cotton swab. The migrated cells were fixed with 4% paraformaldehyde (Beyotime, China), stained with 0.1% crystal violet (Beyotime, China), and quantified. Results are presented as the migration/invasion rate (%) relative to the control. All functional assays were independently repeated three times (*n* = 3).

### Western blot assay

For western blotting, cells were lysed in RIPA buffer (Beyotime, China) with PMSF (Beyotime, China), and nuclear/cytoplasmic fractions were isolated using the NE-PER Kit (Thermo Fisher Scientific, USA). Protein concentrations were measured by BCA assay (Thermo Fisher Scientific, USA). Equal protein amounts were resolved by SDS-PAGE, transferred to PVDF membranes (Millipore, USA), and blocked with 5% non-fat milk. Membranes were probed with primary antibodies overnight at 4 °C, then with HRP-conjugated secondary antibodies. Protein signals were detected using an ECL kit (Millipore, USA) and a chemiluminescence imaging system. Experiments were repeated in triplicate (*n* = 3).

### Luciferase reporter assay

To validate direct targeting of *FZD2* by miR-5584-5p, we used the miRDB database to predict their binding sites. We cloned wild-type (*FZD2*-WT) or mutant (*FZD2*-MUT) sequences into the pmirGLO vector (Promega, USA). Furthermore, to evaluate Wnt/β-catenin transcriptional activity, cells were co-transfected with TOPflash or FOPflash reporter plasmids (containing wild-type or mutated TCF/LEF binding sites, respectively) along with the Renilla luciferase plasmid and the respective miRNA mimics, inhibitors, or overexpression plasmids. Luciferase activity in co-transfected Ishikawa cells was then measured 48 h post-transfection using the Dual-Luciferase^®^ Reporter Assay System (Promega, USA). For the Wnt activity assay, the TOP/FOP ratio was calculated after normalization to Renilla activity. Assays were performed in independent triplicates (*n* = 3).

### Statistical analysis

Statistical analyses were performed using SPSS 26.0 and GraphPad Prism 8.0. All cell-based experiments were performed in triplicate with at least three independent biological replicates, and data are presented as mean ± SD. Clinical data were compared using Student’s t-test or Mann-Whitney U test for continuous variables, and χ² test or Fisher’s exact test for categorical variables. Multi-group comparisons were analyzed by one-way ANOVA with Tukey’s post hoc test. Multivariate logistic regression was used to identify factors independently associated with EM, adjusting for age, BMI, and menstrual cycle phase, with results reported as ORs and 95% CIs. Multicollinearity among variables was assessed using the Variance Inflation Factor (VIF), with a VIF < 5 indicating no significant collinearity. Diagnostic performance was evaluated by ROC curve analysis, presenting AUC with 95% CI, and Youden’s index was used to determine optimal cut-off values. To assess the robustness and potential overfitting of the diagnostic model, a stratified 5-fold cross-validation was performed. The dataset was randomly partitioned into five folds, maintaining the original ratio of EM cases to controls. In each iteration, four folds served as the training set and the remaining fold as the test set. The mean AUC and standard deviation (SD) were calculated across the five iterations to evaluate the model’s generalizability. Correlations were analyzed by Spearman’s rank correlation coefficient. *P* < 0.05 was considered statistically significant.

## Results

### Basic characteristics of the study population

A total of 110 control subjects and 107 patients with EM were included in this study. There were no significant differences in age (*P =* 0.495), BMI (*P =* 0.214), pain score (VAS) (*P =* 0.190), duration of menstrual bleeding (*P =* 0.177), and abortion history (*P =* 0.823) between the two groups. However, the menstrual cycle length of patients in the EM group was significantly shorter than that of the control group (*P =* 0.006), and the proportion of patients with a family history of EM in the EM group was significantly higher than that of the control group (*P =* 0.001, Table 1).

**Table 1:** General information of the study subjects

	Control (*n* = 110)	EM (*n* = 107)	*P* value
Age (years)	33.40 ± 5.84	32.89 ± 5.16	0.495
BMI (kg/m^2^)	24.79 ± 4.70	24.12 ± 3.15	0.214
Pain Score (VAS)	4.42 ± 1.83	4.78 ± 2.16	0.190
Menstrual cycle length (days)	31.68 ± 7.50	28.93 ± 7.18	0.006
Duration of bleeding menstrual (days)	6.25 ± 1.86	5.93 ± 1.61	0.177
History of abortion (Yes/No)	12 / 98	10 / 97	0.823
Family history of endometriosis (Yes/No)	25 / 85	47 / 60	0.001
rASRM classification			
I - II		69	
III - IV		38	

*EM* Endometriosis, *BMI* Body Mass Index, *VAS* Visual Analog Scale, *rASRM* Revised American Society for Reproductive Medicine classification

### Endometriosis: logistic regression and miR-5584-5p diagnostic assessment

To evaluate factors associated with EM risk, logistic regression analysis was performed. As shown in Table 2, higher miR-5584-5p expression level (OR = 0.043, 95% CI: 0.020–0.092, *P* < 0.001) and longer menstrual cycle (OR = 0.339, 95% CI: 0.159–0.721, *P =* 0.005) were associated with reduced odds of EM, while family history of EM was associated with increased odds (OR = 3.119, 95% CI: 1.366–7.121, *P =* 0.007). Collinearity diagnostics confirmed model stability, with all VIF values ranging from 1.012 to 1.051, indicating the absence of significant multicollinearity. It is important to note that miR-5584-5p was entered into the model as a continuous variable based on its relative expression level; thus, the low OR (0.043) reflects the mathematical odds change per one-unit increase in this specific scale. Nevertheless, this OR estimate is derived from a single-center cross-sectional cohort and should be interpreted with caution, requiring further validation in larger and more diverse populations.

**Table 2:** Logistic analysis of risk factors for EM

	OR	95% CI	*P* value
miR-5584-5p	0.043	0.020–0.092	< 0.001
Age (years)	0.733	0.347–1.547	0.415
BMI (kg/m^2^)	0.731	0.345–1.546	0.412
Pain Score (VAS)	1.944	0.901–4.197	0.090
Menstrual cycle length (days)	0.339	0.159–0.721	0.005
Duration of bleeding menstrual (days)	0.565	0.266–1.202	0.138
History of abortion (Yes/No)	1.337	0.382–4.687	0.650
Family history of endometriosis (Yes/No)	3.119	1.366–7.121	0.007

*EM* Endometriosis, *BMI* Body Mass Index, *VAS* Visual Analog Scale, *rASRM* Revised American Society for Reproductive Medicine classification

**Fig. 1:**
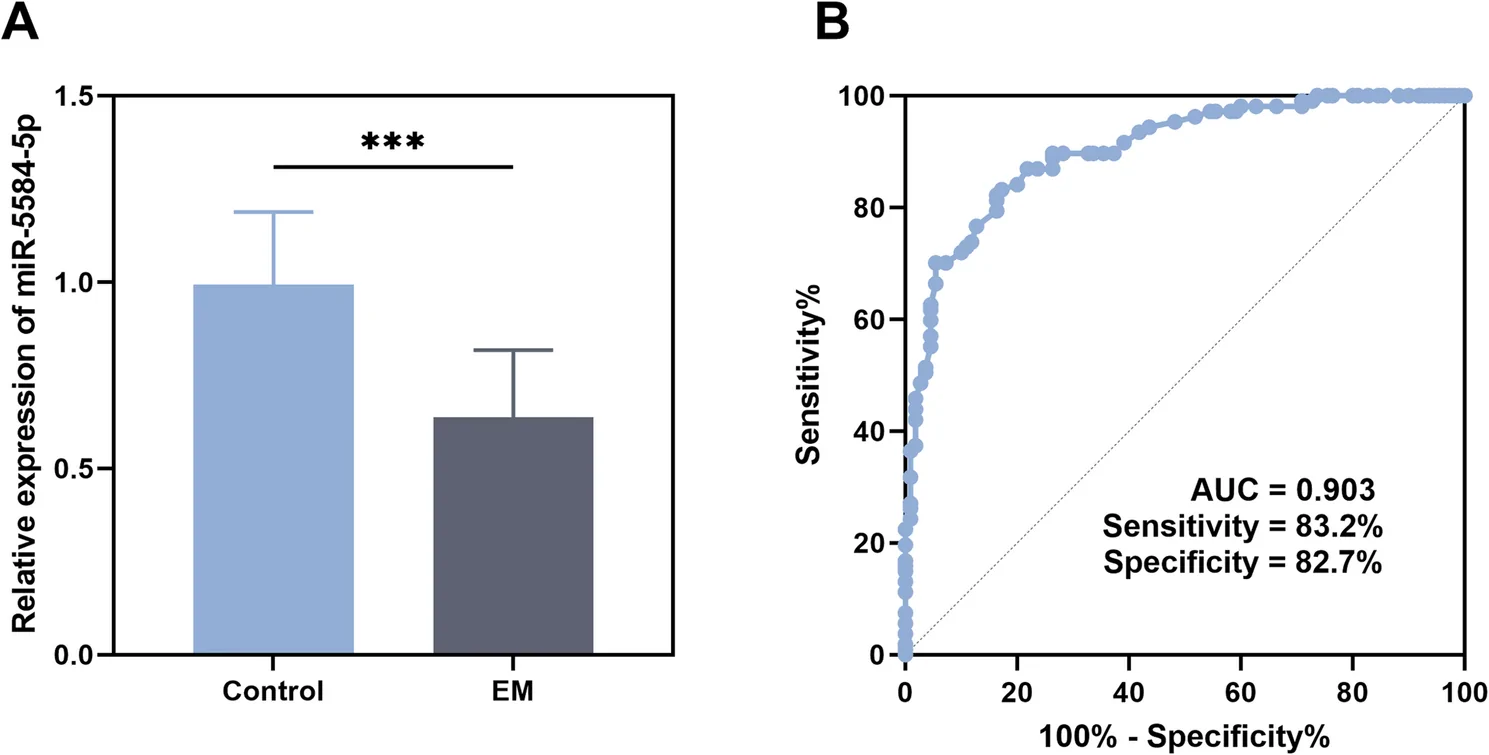
Clinical significance of miR-5584-5p in EM. miR-5584-5p expression in EM and Control serum (**A**), and its diagnostic performance evaluated by ROC curve **B**. EM: endometriosis; ROC: receiver operating characteristic. ***P < 0.001

Serum levels of miR-5584-5p were significantly lower in EM patients than in controls (*P* < 0.001, Fig. 1A). ROC analysis showed that miR-5584-5p had an initial apparent AUC of 0.903 (95% CI: 0.862–0.944) for distinguishing EM patients from controls, with 83.2% sensitivity and 82.7% specificity (Fig. 1B). Furthermore, the internal stability of this diagnostic performance was confirmed via stratified 5-fold cross-validation, which yielded a mean AUC of 0.901 ± 0.075 (individual fold AUCs: 0.924, 0.980, 0.844, 0.802, and 0.953; Supplemental Table 1). These results indicate that the high diagnostic accuracy of miR-5584-5p is consistent and not a result of overfitting to a specific data subset.

### Effects of miR-5584-5p on Ishikawa cell functions

**Fig. 2:**
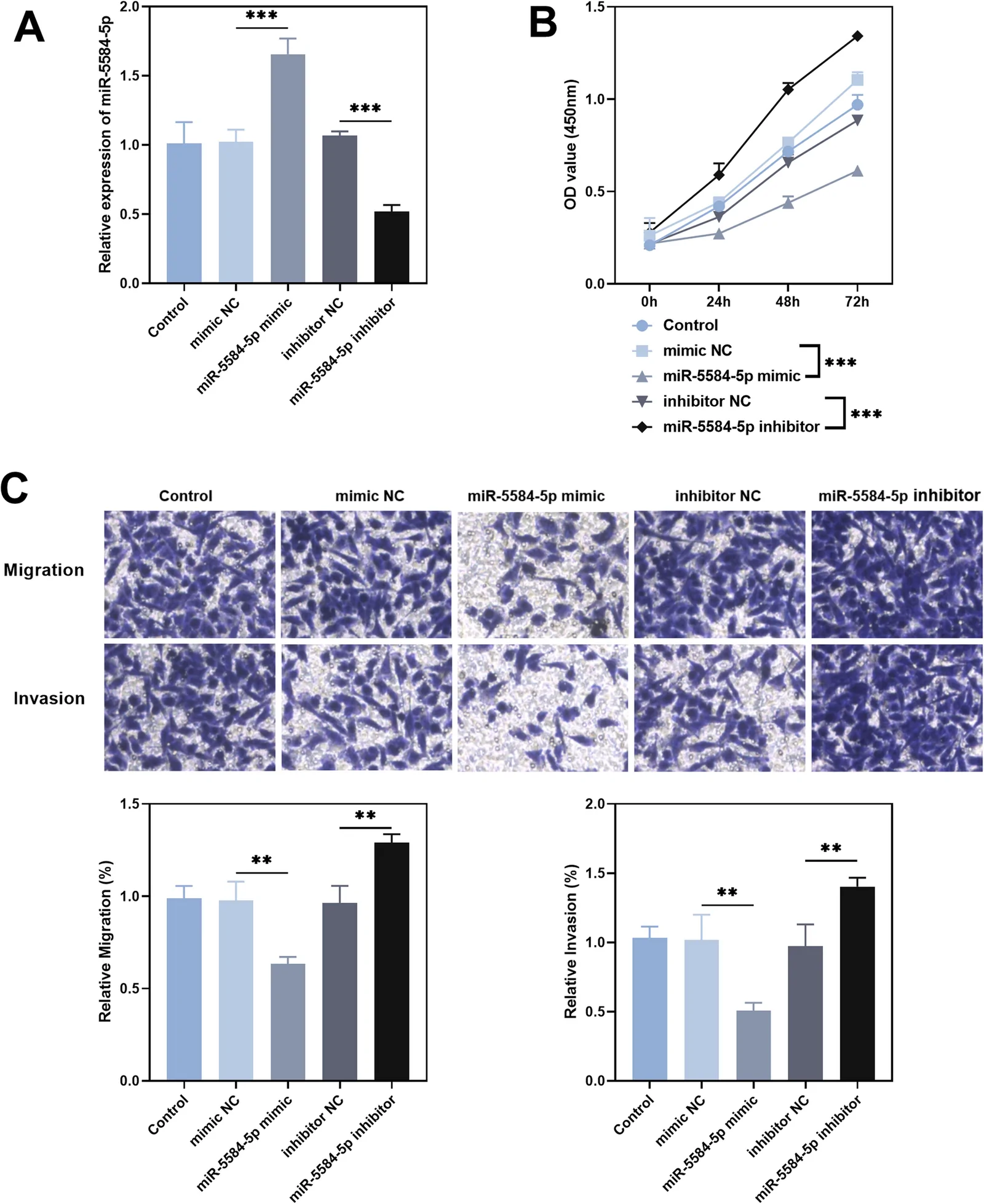
Functional impacts of miR-5584-5p on Ishikawa cell phenotypes. Transfection efficacy (**A**), cell proliferation (**B**), and cell migration and invasion capacities **C**. CCK-8: Cell Counting Kit-8. **P < 0.01, ***P < 0.001

Functional analysis of miR-5584-5p was performed in Ishikawa cells using gain- and loss-of-function approaches. Transfection of miR-5584-5p mimic significantly increased miR-5584-5p levels, while transfection of the inhibitor decreased its expression (*P* < 0.001, Fig. 2A). Functionally, CCK-8 and Transwell assays demonstrated that miR-5584-5p overexpression significantly suppressed cell proliferation, migration, and invasion (Fig. 2B-C). Conversely, miR-5584-5p inhibition promoted these malignant phenotypes (*P* < 0.001).

### Effects of miR-5584-5p on Wnt/β-catenin-EMT axis

**Fig. 3:**
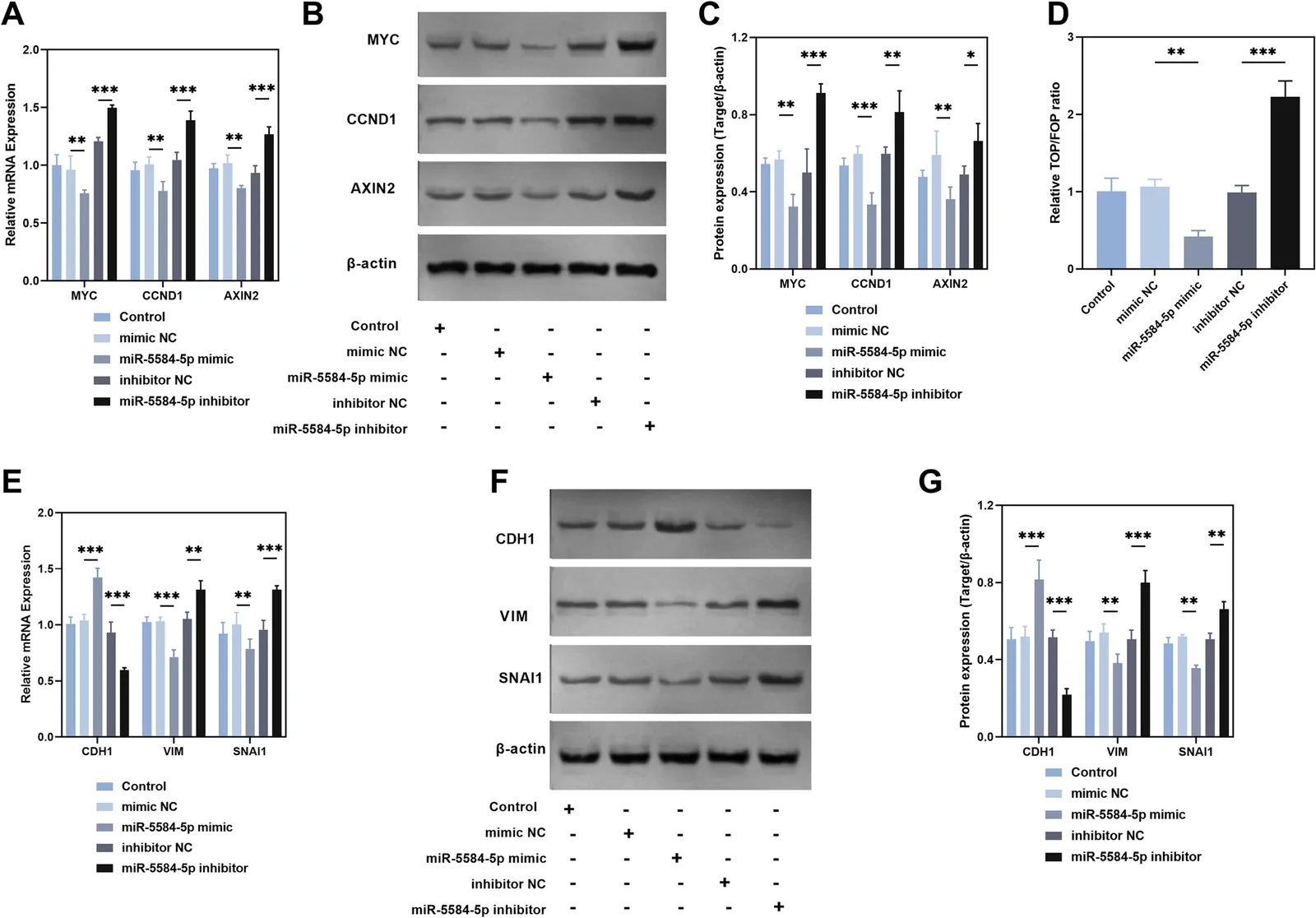
miR-5584-5p modulates the Wnt/β-catenin EMT axis. mRNA (**A**) and protein (**B**, **C**) levels of Wnt/β-catenin targets (*MYC*, *CCND1*, *AXIN2*). Wnt/β-catenin transcriptional activity (**D**). mRNA (**E**) and protein (**F**, **G**) levels of EMT markers (*CDH1*, *VIM*, *SNAI1*). EMT: epithelial-mesenchymal transition; mRNA: messenger RNA. *P < 0.05, **P < 0.01, ***P < 0.001

qRT-PCR and Western blot analyses revealed that miR-5584-5p overexpression significantly reduced both mRNA and protein levels of Wnt/β-catenin target genes (*MYC*, *CCND1*, *AXIN2*) (Fig. 3A-C). Furthermore, TOP/FOP flash reporter assays confirmed that miR-5584-5p overexpression significantly suppressed the relative TOP/FOP ratio compared to the control (*P* < 0.01), whereas miR-5584-5p inhibition significantly increased it (*P* < 0.001, Fig. 3D). Regarding the EMT markers, miR-5584-5p overexpression increased the epithelial marker *CDH1* and decreased mesenchymal markers (*VIM*, *SNAI1*) at both transcript and protein levels, with opposing effects observed upon miR-5584-5p inhibition *(P* < 0.001, Fig. 3E-G).

### miR-5584-5p directly targets FZD2

**Fig. 4:**
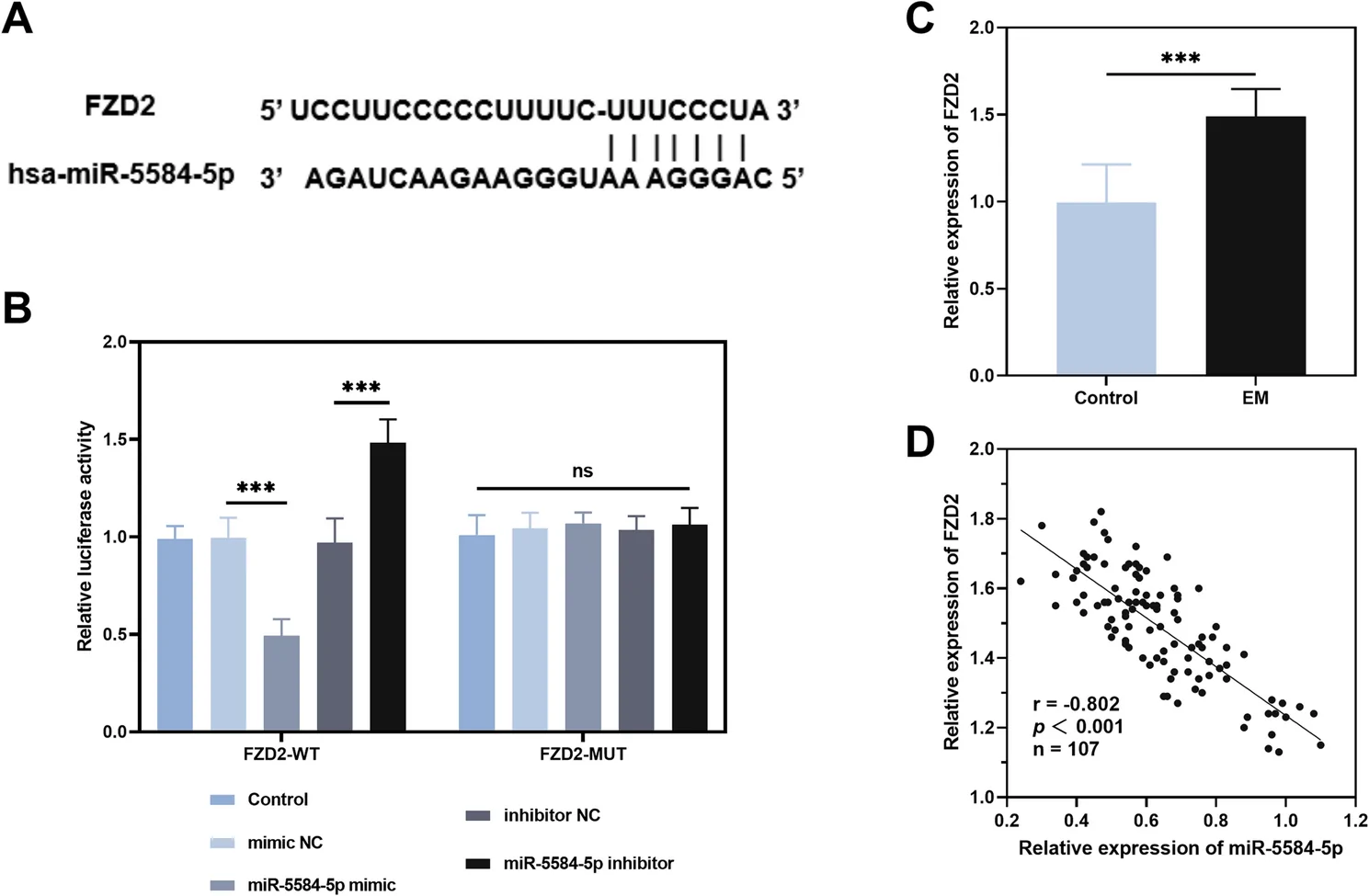
miR-5584-5p directly targets *FZD2* expression. Predicted binding site (**A**) and luciferase reporter assay (**B**) for miR-5584-5p and FZD2. FZD2 mRNA levels in clinical serum (**C**), and its correlation with miR-5584-5p in EM patients **D**. UTR: untranslated region; ns: not significant, ***P < 0.001

To explore the downstream targets of miR-5584-5p, bioinformatics analysis predicted *FZD2* as a potential direct target gene, with an identified binding site between them (Fig. 4A). Dual-luciferase reporter assays showed that miR-5584-5p mimic significantly reduced the luciferase activity of the wild-type *FZD2* 3’UTR reporter, while the mutant reporter was unaffected; miR-5584-5p inhibitor had the opposite effect (*P* < 0.001; Fig. 4B), confirming direct targeting.

Subsequently, we detected *FZD2* expression at both the tissue and cellular levels. As shown in Fig. 4C, compared with the control group, the mRNA expression level of *FZD2* in the serum of EM patients was significantly upregulated (*P* < 0.001). Furthermore, Spearman’s correlation analysis revealed a significant negative correlation between serum miR-5584-5p and *FZD2* expression in EM patients (Fig. 4D).

### FZD2 overexpression partially reverses the effects of miR-5584-5p

**Fig. 5:**
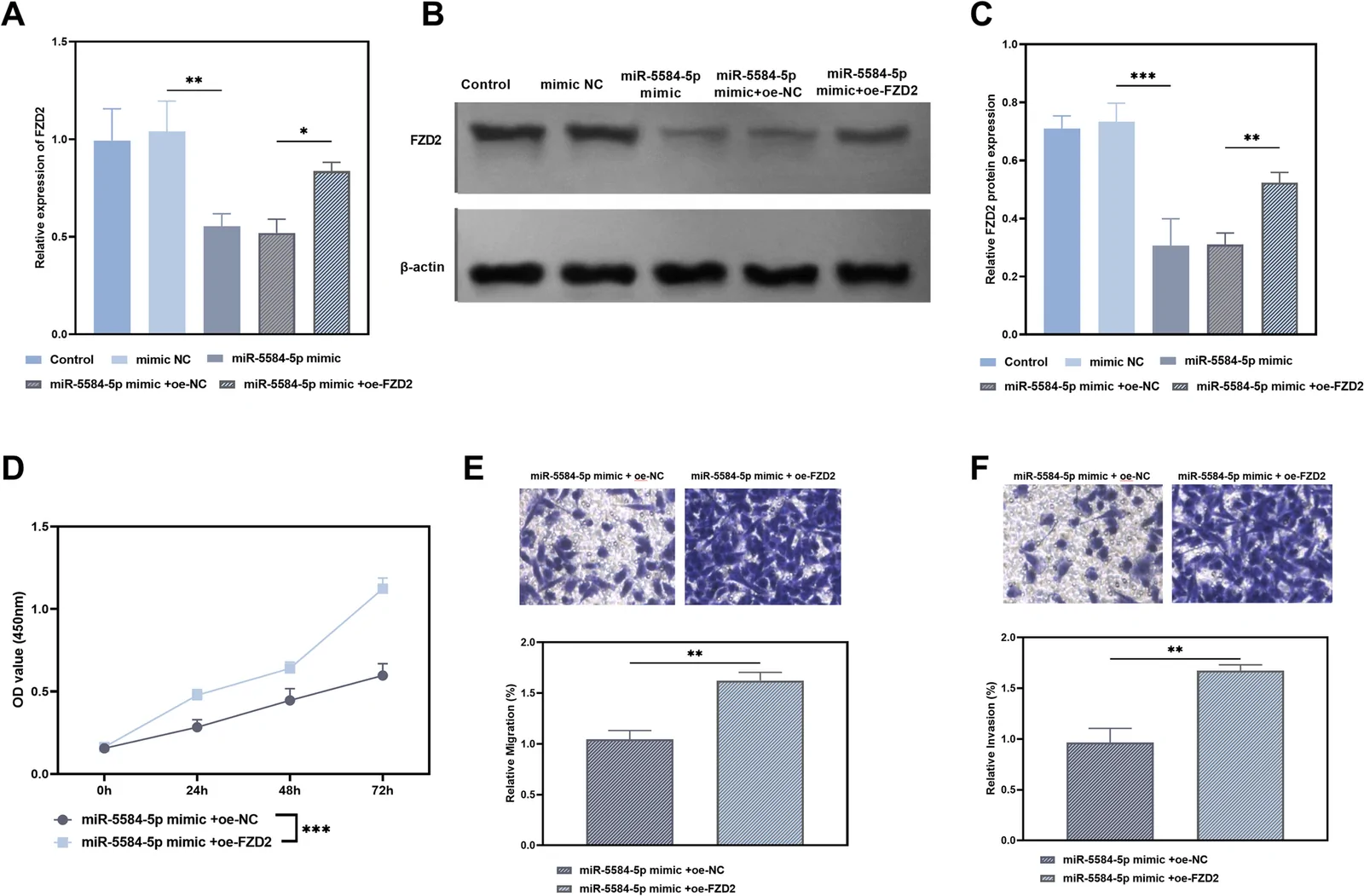
*FZD2* overexpression reverses the phenotypic effects of miR-5584-5p. Validation of *FZD2* mRNA (**A**) and protein (**B**, **C**) overexpression. Effects of *FZD2* restoration on cell proliferation (**D**), migration (**E**), and invasion **F**.*P < 0.05, **P < 0.01, ***P < 0.001

To verify whether miR-5584-5p functions by targeting *FZD2*, we conducted rescue experiments by overexpressing miR-5584-5p while simultaneously introducing an *FZD2* overexpression plasmid. As shown in Fig. 5A, compared with cells co-transfected with the empty control (miR-5584-5p mimic + OE-NC), co-transfection with *FZD2* (miR-5584-5p mimic + OE-*FZD2*) significantly restored the mRNA expression level of *FZD2* (*P* < 0.001). This was also confirmed at the protein level by Western blot analysis (Fig. 5B-C), indicating the effectiveness of the rescue experimental system.

Functionally, *FZD2* overexpression partially reversed the effects of miR-5584-5p mimic on Ishikawa cell proliferation (Fig. 5D), migration (Fig. 5E), and invasion (Fig. 5F).

**Fig. 6:**
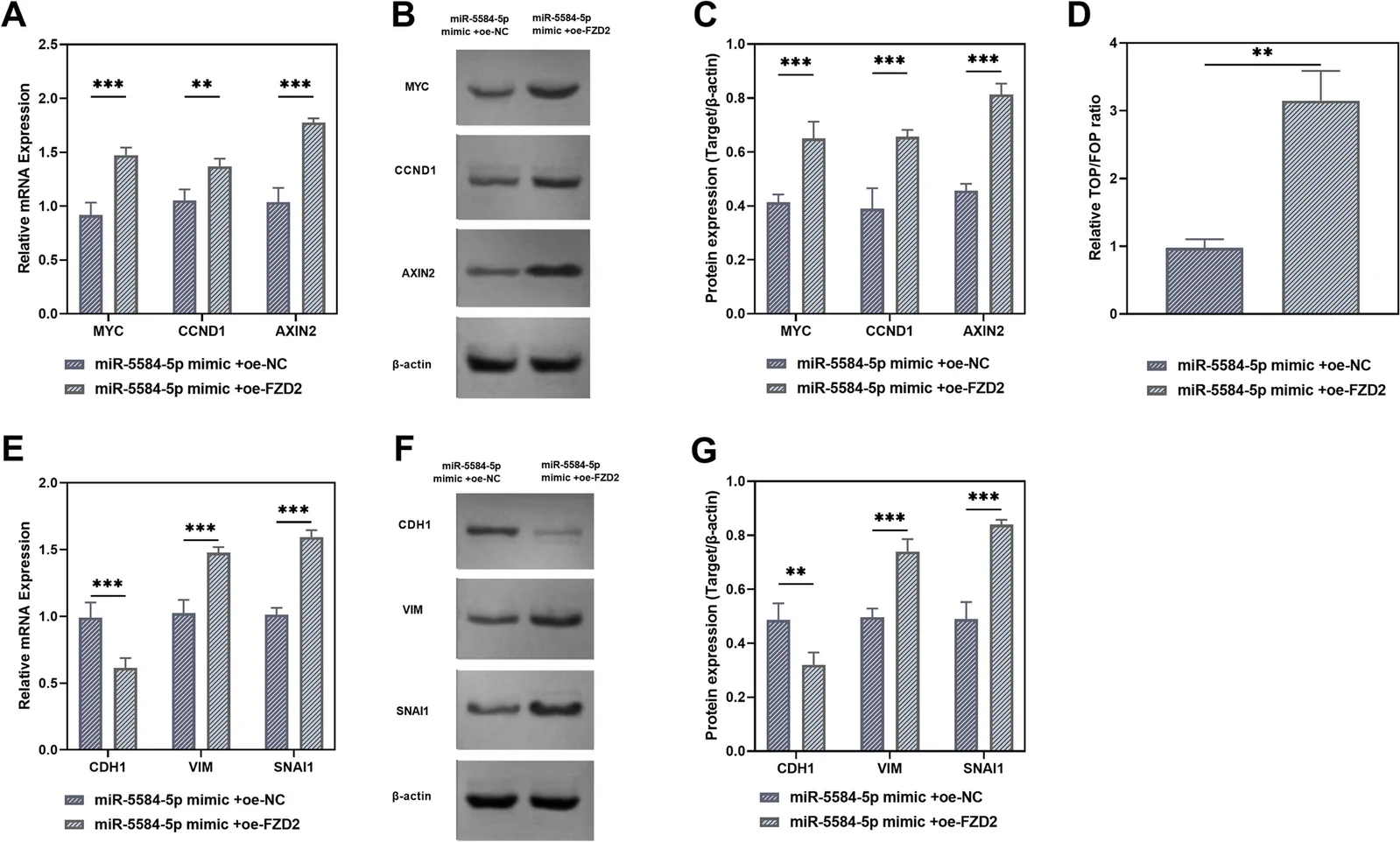
*FZD2* mediates miR-5584-5p-induced modulation of Wnt/β-catenin-EMT signaling. Restoration of mRNA (**A**) and protein (**B**, **C**) levels of Wnt/β-catenin targets. Rescue of Wnt/β-catenin transcriptional activity **D**. Recovery of mRNA (**E**) and protein (**F**, **G**) expression of EMT markers. **P < 0.01, ***P < 0.001

Mechanistically, reintroduction of *FZD2* was associated with increased mRNA and protein levels of Wnt/β-catenin target genes (*MYC*, *CCND1*, *AXIN2*) (Fig. 6A-C). Consistent with these findings, the TOP/FOP reporter assay demonstrated that the reintroduction of *FZD2* significantly rescued the relative TOP/FOP ratio (Fig. 6D, *P* < 0.01), effectively restoring the Wnt/β-catenin transcriptional activity that was suppressed by the miR-5584-5p mimic. Furthermore, *FZD2* overexpression successfully reversed EMT marker expression, restoring *CDH1* and increasing *VIM*/*SNAI1* at both the mRNA (Fig. 6E) and protein levels (Fig. 6F-G).

## Discussion

The regulatory role of miRNAs in the pathogenesis and diagnosis of EM has gained growing recognition, particularly in shaping the immune microenvironment and regulating endometrial cell migration [15–17]. In this study, serum analysis of 110 dysmenorrhea patients without EM and 107 EM patients revealed significant downregulation of miR-5584-5p in the EM group. ROC analysis demonstrated high diagnostic accuracy (AUC = 0.903), with 83.2% sensitivity and 82.7% specificity. The reliability of this performance was further confirmed using stratified 5-fold cross-validation, maintaining a high mean AUC of 0.901 (SD = 0.075). This internal validation significantly mitigates concerns regarding potential overfitting.

However, several caveats exist. The control group consisted of women with dysmenorrhea rather than asymptomatic healthy individuals. While this reflects clinical diagnostic realities, dysmenorrhea itself may influence miRNA expression. Furthermore, this diagnostic performance was derived from a single cohort without external validation, meaning the extreme odds ratio (OR = 0.043) must be interpreted cautiously. Nevertheless, the non-invasive detection of serum miR-5584-5p shows great potential as a diagnostic biomarker for EM, warranting further investigation in multi-center studies.

In the complex pathological process of EM, abnormal cell proliferation constitutes the basis for the formation of ectopic lesions [18, 19]. In this study, miR-5584-5p overexpression was associated with reduced proliferation of Ishikawa cells, while miR-5584-5p inhibition had opposing effects. The enhanced migratory and invasive capabilities of cells represent a core aspect of disease progression [20, 21], and we observed that miR-5584-5p overexpression was associated with decreased migration and invasion. These findings suggest that miR-5584-5p may play a role in regulating endometrial epithelial cell behaviors relevant to EM pathogenesis.

To further explore the potential mechanisms underlying these phenotypic changes, we examined the canonical Wnt/β-catenin signaling pathway, which is known to promote cell cycle progression and EMT [22, 23]. In Ishikawa cells, miR-5584-5p overexpression was associated with reduced mRNA and protein levels of downstream targets that drive cell cycle progression (*MYC*, *CCND1*, and *AXIN2*) [24–26], further supporting the modulation of Wnt/β-catenin signaling. Crucially, our TOP/FOP flash reporter assays provided direct evidence that miR-5584-5p significantly suppresses actual Wnt/β-catenin transcriptional activity. Furthermore, miR-5584-5p overexpression was associated with increased expression of the epithelial marker *CDH1* [27] and decreased expression of the mesenchymal markers *VIM* and the transcription factor *SNAI1* [28] at both mRNA and protein levels. These findings suggest that miR-5584-5p may modulate the Wnt/β-catenin-EMT axis in Ishikawa cells, providing a potential mechanistic link to its observed effects on cell migration and invasion.

FZD2, a member of the Frizzled receptor family involved in Wnt/β-catenin signaling [29], has been reported to promote cell migration, invasion, and EMT in cancer models [30]. In this study, *FZD2* mRNA levels were elevated in serum from EM patients compared to controls, consistent with previous observations [31]. An inverse correlation between miR-5584-5p and *FZD2* expression was observed at both mRNA and protein levels in Ishikawa cells. Luciferase reporter assays confirmed *FZD2* as a direct target of miR-5584-5p, and rescue experiments showed that overexpression of *FZD2* partially reversed the effects of miR-5584-5p on cell proliferation, migration, invasion, and on Wnt/β-catenin-EMT-related gene and protein expression. These findings suggest that *FZD2* mediates, at least in part, the effects of miR-5584-5p in this cell model. Based on these in vitro observations, the miR-5584-5p/*FZD2* axis may influence endometrial epithelial cell behaviors relevant to EM pathogenesis through modulation of cell cycle-related genes and EMT-associated markers. However, the extent to which this axis contributes to EM progression in vivo requires further investigation.

Several limitations of this study should be acknowledged. First, the clinical control group consisted of women with dysmenorrhea rather than healthy individuals, which may influence miRNA expression. Furthermore, the single-center design and the extreme odds ratio (OR = 0.043) require cautious interpretation. Although the 5-fold cross-validation (mean AUC = 0.901) supports internal robustness, external validation in independent multi-center cohorts remains essential to confirm clinical utility. Second, all in vitro functional experiments utilized Ishikawa cells. Although these are standard for modeling endometrial epithelial biology, their malignant origin and the lack of stromal or immune components mean they cannot fully recapitulate benign EM lesions. Third, while we confirmed Wnt/β-catenin modulation, we did not evaluate β-catenin nuclear translocation or utilize pharmacological Wnt inhibitors. Additionally, miRNAs typically regulate multiple genes, meaning targets other than *FZD2* might also contribute to the observed phenotypes. Fourth, the absence of an in vivo animal model limits the definitive causal assessment of the miR-5584-5p/*FZD2* axis in lesion establishment and progression. Finally, EM pathogenesis involves complex immune and hormonal interactions beyond the Wnt/β-catenin-EMT axis. The relationship between *FZD2* signaling in EM versus cancer, along with the upstream mechanisms causing miR-5584-5p downregulation, remains to be explored. Despite these limitations, this study provides initial evidence that miR-5584-5p is downregulated in EM serum and may contribute to regulating endometrial epithelial cell behavior by targeting *FZD2*, offering a foundation for future research using more physiologically relevant models and larger cohorts.

In summary, this study provides initial evidence that serum miR-5584-5p is downregulated in EM patients and may have potential as a diagnostic aid. In vitro findings using Ishikawa cells suggest that miR-5584-5p may contribute to the regulation of endometrial epithelial cell behaviors through targeting *FZD2* and modulating the Wnt/β-catenin-EMT axis. These findings offer a foundation for future research using more physiologically relevant models and larger clinical cohorts to further evaluate the diagnostic and therapeutic potential of miR-5584-5p in endometriosis.

## Conclusion

This study provides initial evidence that serum miR-5584-5p is downregulated in EM patients and may have potential as a diagnostic aid in this population. In vitro findings using Ishikawa cells suggest that miR-5584-5p may contribute to the regulation of endometrial epithelial cell behaviors—including proliferation, migration, and invasion—through targeting *FZD2* and modulating the Wnt/β-catenin-EMT axis. Further validation in primary cells, animal models, and multi-center cohorts is warranted to confirm these findings and explore their translational potential.

## Supplementary Information

Supplementary Material 1.

## Data Availability

The datasets used and/or analysed during the current study are available from the corresponding author on reasonable request.
