# Supplementary material for: Diagnostic and mechanistic roles of miR-5584-5p in endometriosis via the FZD2-mediated Wnt/β-catenin EMT axis
Source: Hereditas. 2026 Aug 21;163:101. doi: 10.1186/s41065-026-00732-4 (PMC13628742; doi:10.1186/s41065-026-00732-4)
Supplement: Supplementary file 1 [file 41065_2026_732_MOESM1_ESM.docx]

**Supplemental Table 1** Detailed results of 5-fold cross-validation

| Fold | AUC | 95% CI | *P* value |
| --- | --- | --- | --- |
| 1 | 0.924 | 0.841-1.000 | <0.001 |
| 2 | 0.980 | 0.950-1.000 | <0.001 |
| 3 | 0.844 | 0.722-0.966 | <0.001 |
| 4 | 0.802 | 0.674-0.930 | 0.001 |
| 5 | 0.953 | 0.896-1.000 | <0.001 |
| Mean ± SD | 0.901 ± 0.075 | / | / |

AUC, area under the curve; 95% CI, 95% confidence interval; SD, standard deviation.
